# Intercellular communication involving macrophages at the maternal-fetal interface may be a pivotal mechanism of URSA: a novel discovery from transcriptomic data

**DOI:** 10.3389/fendo.2023.973930

**Published:** 2023-05-17

**Authors:** Xiaoxuan Zhao, Yuepeng Jiang, Shiling Luo, Yang Zhao, Hongli Zhao

**Affiliations:** ^1^Department of Traditional Chinese Medicine (TCM) Gynecology, Hangzhou Hospital of Traditional Chinese Medicine Affiliated to Zhejiang Chinese Medical University, Hangzhou, China; ^2^College of Pharmaceutical Science, Zhejiang Chinese Medical University, Hangzhou, China; ^3^The First Clinical Medical College of Nanjing University of Chinese Medicine, Nanjing, China

**Keywords:** Unexplained recurrent spontaneous abortion, maternal-fetal interface, macrophage, decidual stromal cells, placenta, intercellular communication

## Abstract

Unexplained recurrent spontaneous abortion (URSA) is a severe challenge to reproductive females worldwide, and its etiology and pathogenesis have not yet been fully clarified. Abnormal intercellular communication between macrophages (Mφ) and decidual stromal cells (DSCs) or trophoblasts has been supposed to be the key to URSA. However, the exact molecular mechanisms in the crosstalk are not yet well understood. This study aimed to explore the potential molecule mechanism that may be involved in the communication between Mφ and DSC or trophoblast cells and determine their diagnostic characteristics by using the integrated research strategy of bioinformatics analysis, machine learning and experiments. First, microarrays of decidual tissue (GSE26787, GSE165004) and placenta tissue (GSE22490) in patients with URSA, as well as microarrays involving induced decidualization (GSE94644) and macrophage polarization *in vitro* (GSE30595) were derived from the gene expression omnibus (GEO) database. And 721 decidua-differentially expressed genes (DEGs), 613 placenta-DEGs, 510 Mφ polarization DEGs were obtained in URSA by differential expression analysis. Then, the protein-protein interaction (PPI) network was constructed, and the hub genes were identified by CytoHubba in Cytoscape software and validated by real-time PCR assay. Subsequently, immune enrichment analysis on decidua-DEGs and placenta-DEGs by ClueGO verified their regulation effects on Mφ. Besides, functional enrichment analysis was performed on Mφ polarization DEGs and the essential module genes derived from the weighted gene co-expression network analysis (WGCNA) to uncover the biological function that were related to abnormal polarization of Mφ. Furthermore, we screened out 29, 43 and 22 secreted protein-encoding genes from DSC-DEGs, placenta-DEGs and Mφ polarization DEGs, respectively. Besides, the hub secreted-protein-encoding genes were screened by CytoHubba. Moreover, we conducted functional enrichment analysis on these genes. And spearman correlation analysis between hub secreted-protein-encoding genes from donor cells and hub genes in recipient cells was performed to further understand the molecular mechanism of intercellular communication further. Moreover, signature genes with diagnostic value were screened from secreted protein-encoding genes by machine learning and validated by immunofluorescence co-localization analysis with clinical samples. Finally, three biomarkers of DSCs (FGF9, IL1R2, NID2) and three biomarkers of Mφ (CFB, NID2, CXCL11) were obtained. In conclusion, this project provides new ideas for understanding the mechanism regulatory network of intercellular communication involving macrophages at the maternal-fetal interface of URSA. Also, it provides innovative insights for the diagnosis and treatment of URSA.

## 1 Introduction

Recurrent spontaneous abortion (RSA) is defined as two or more consecutive miscarriages before 20 weeks of gestation with the same partner (1, 2). Approximately 1-5% reproductive women are afflicted with RSA, and 40-50% of cases remain unclear, which is defined as unexplained recurrent spontaneous abortion (URSA) (3). Accumulated evidence has profiled that disturbed regulatory mechanisms in the maternal-fetal interface are mainly responsible for URSA (4). Maternal-fetal interface is a crucial site for mutual recognition and information transfer between mothers and fetus, which mainly comprises decidual stromal cells (DSCs), decidual immune cells (DICs) and trophoblasts (5). DSCs are differentiated from endometrial stromal cells, i.e., decidualization, under the influence of hormones or embryo implantation signals (6). The decidualized phenotype contributes to precisely timed adaptations in endometrium for fetal growth (7). Trophoblasts, the main component of the placenta, are responsible for invading and reshaping the endometrial stroma and blood vessels in early pregnancy. What’s more, multiple researchers have suggested that the cellular abundance, phenotypes, and functions of DICs can be finely adjusted by DSCs and trophoblasts, the active contributors to the homeostatic and tolerogenic properties of maternal-fetal interface (8, 9). In turn, DICs can mediate maternal-derived DSC and fetal-derived trophoblasts (9). Once the complex and sophisticated cell-cell crosstalk is disrupted, recurrent embryo loss may occur. Therefore, exploring the mechanism of intercellular communication between DSCs, trophoblasts and DICs is conducive to further deciphering the pathological mechanisms of URSA.

Currently, various investigations have focused on the role of Mφ in intercellular communication at the maternal-fetal interface in URSA. As the second largest leukocytes at the maternal-fetal interface, Mφ account for 20-30% of DICs, and plays pivotal roles in regulating trophoblast invasion, remodeling spiral arteries, and maintenance of immune homeostasis (10, 11). Based on cellular phenotype and function, Mφ can be broadly categorized into classically activated subtypes (M1) and alternatively activated subtypes (M2) (12, 13), in which M1-polarized Mφ can efficiently clear antigens and switch T lymphocyte responses to T helper-1 (Th1) immune responses, thus inducing embryo rejection; while M2-polarized Mφ exhibit immunosuppressive properties and contribute to tissue remodeling, promoting Th2 immune response and maternal immune tolerance to embryos (14). Besides, neighboring cells, such as DSCs and trophoblasts, are thought to be critical in regulating Mφ polarization. Furthermore, recent studies have shed light on the dysregulation of DSCs as well as trophoblasts on macrophage polarization in URSA (15–17). However, the results are heterogeneous, mainly because traditional molecular biology methods are insufficient to provide a comprehensive landscape of molecule mechanism on intercellular communication. Nowadays, the revolutionary development of microarray techniques and bioinformatics make up for the current shortcomings and greatly facilitate in-depth insights into the characterization and pathogenesis of disease from both molecular and system levels.

**Figure 1:**
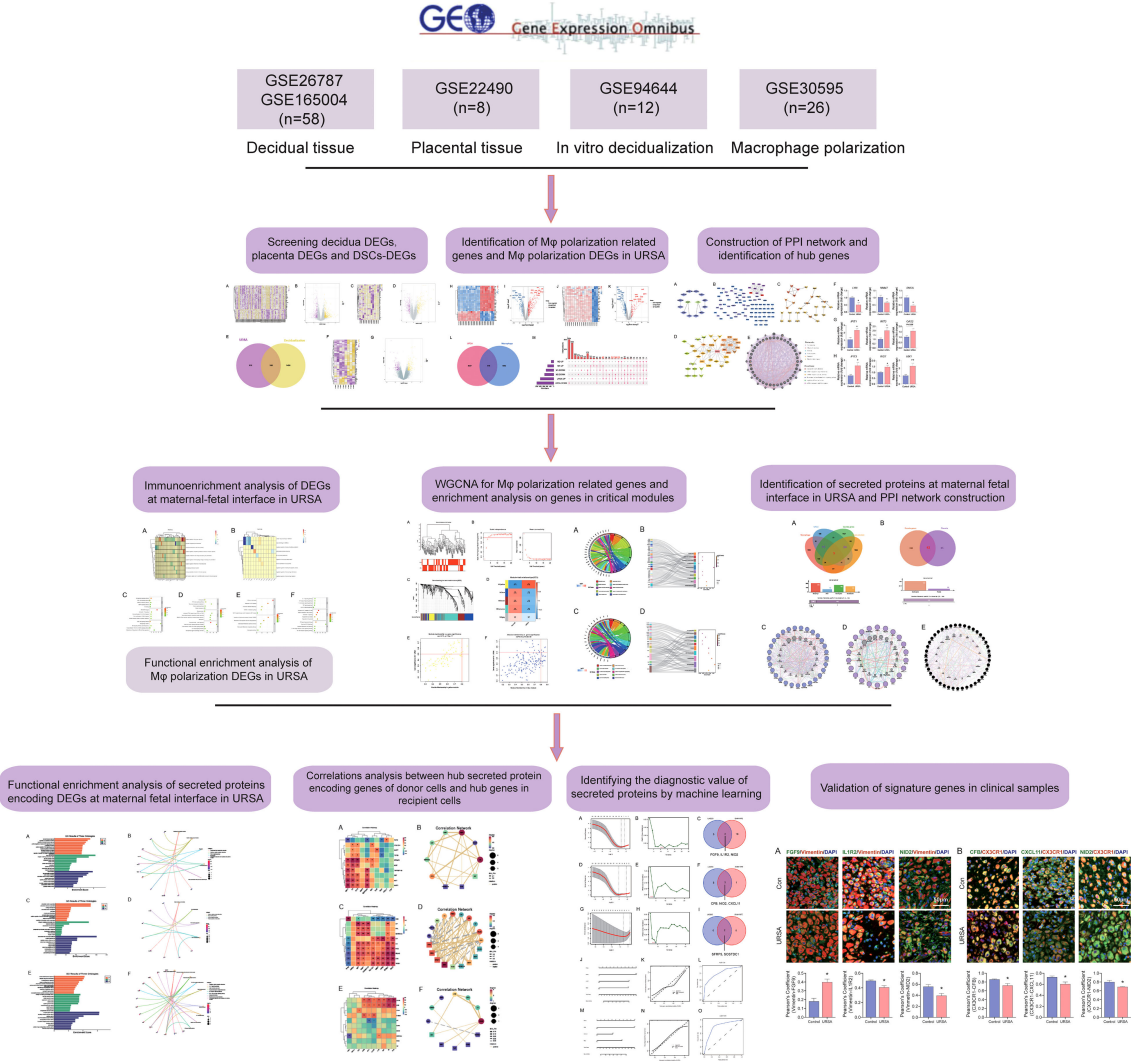
The work flow chart of this study.

In this study, we integrated and analyzed microarrays involving decidua and placental tissue of URSA, along with the mRNA expression profiles of *in vitro* decidualization and Mφ polarization from the gene expression omnibus (GEO) database, to obtain differentially expressed genes (DEGs) derived from decidua, placenta, and decidual stromal cells (DSCs) as well as DEGs related to Mφ polarization in URSA. We then performed protein-protein interaction (PPI) network analysis, hub gene screening and functional analysis by GeneMANIA for DSC-derived DEGs, placenta-derived DEGs and macrophage polarization related DEGs in RSA, respectively. After that, we further performed immune enrichment analysis on decidual and placental-derived DEGs by ClueGO to identify their regulation effects on Mφ. Moreover, functional enrichment analysis was conducted on Mφ polarization related DEGs as well as critical modules of macrophage polarization-related genes through weighted gene co-expression network analysis (WGCNA) to uncover the abnormal molecular mechanisms of Mφ polarization in URSA and the intercellular communication in which these molecules were involved in. Furthermore, we creatively focused on screening secreted protein-encoding genes from the above DEGs and performed PPI network analysis, hub gene screening and functional enrichment analysis on these secreted protein-encoding genes. In addition, we performed spearman correlation analysis on secreted protein-encoding genes and hub genes in receptor cell so as to further understand intercellular communication. Last but not least, biomarkers from secreted proteins were screened by machine learning and experiments based on clinical samples. Herein, our study deciphers the molecular mechanism of intercellular communication at the maternal-fetal interface of URSA, provides ideas for novel diagnostic strategies and targeted therapies for URSA, and also lays the foundation for subsequent studies. The workflow chart of the study is shown in **Figure 1**.

## 2 Materials and methods

### 2.1 Searching and screening microarray data

All the microarray datasets were retrieved from the GEO database (https://www.ncbi.nlm.nih.gov/geo/) (18). Firstly, GSE26787 and GSE165004 that respectively, involved the mRNA expression profiles of decidual tissues, and GSE22490 involved the mRNA expression profiles of villus tissues from URSA and health control samples were included, in which patients with URSA experienced at least two miscarriages, and health control had at least one live birth without a history of adverse pregnancy. Besides, dataset of GSE30595 was included, which involved mRNA expression profiles of Mφ polarization from M0 macrophage to M1 and M2 types *in vitro*. Finally, the dataset of GSE94644, which identified the transcription profiles of induced decidualization, was downloaded. The detailed information on each microarray was shown in **Table 1**.

**Table 1:** The basic information of included dataset.

GSE No.	No. of samples	Platform	Description	Country	Character
GSE26787	5 vs. 5	Affymetrix Human Genome U133 Plus 2. 0 Array	Endometrial biopsy was performed in non conceptional cycle in the middle luteal phase of RPL and healthy fertile women (Controls).	France	URSA decidua
GSE165004	24 vs. 24	Agilent-039494 SurePrint G3 Human GE v2 8x60K Microarray 039381	The midsecretory phase endometria of patients with recurrent pregnancy losses (RPL) by comparing with the endometria of healthy fertile women (Controls).	Turkey	URSA decidua
GSE94644	7 vs. 5	Agilent-026652 Whole Human Genome Microarray 4x44K v2	Human Endometrial Stromal Cells (hESCs) obtained from normal pregnancy.	USA	Decidualization
GSE22490	5 vs. 3	[HG-U133_Plus_2] Affymetrix Human Genome U133 Plus 2.0 Array	10 gene arrays, 6 in control group and 4 in cases. (Samples less than 12 weeks were selected in this study)	Estonia	URSA placenta
GSE30595	M0:14 M1:9 M2:3	Agilent-028100 HumanMacroTreg	Comparative expression analysis of CD14+ cells isolated from decidua from pregnant women, CD14+ cells isolated from non-pregnant women and different types of in vitro-generated macrophages.	Germany	Macrophage polarization

### 2.2 Data preprocessing, normalizing and screening of DEGs

The raw data derived from GEO were preprocessed and normalized by R statistical software (version 4.1.2, https://www.r-project.org/) and Bioconductor analysis tools (http://www.bioconductor.org/). The robust multiarray averaging (RMA) and FPKM (Fragments Per Kilobase per Million) algorithms were utilized to implement background correction and quantile normalization. And the effect of batch correction was verified with the “ggplot2” package in R software, as shown in (**Supplementary Figure 1**). After that, DEGs were screened out by using the “limma” package, with the filtering condition of *P* value < 0.05 and |log2FC| >0.5. Besides, the volcano map and heat map of DEGs were respectively drawn by the “ggplot2” package and the “pheatmap” package. Then, Venn diagram was conducted to screen out the overlapped genes through the online software: jvenn (http://www.bioinformatics.com.cn/) (19).

### 2.3 Construction of PPI network and identification of hub genes

The STRING database (https://string-db.org/) was applied to perform PPI network analysis (20). CytoHubba, a Cytoscape software (version 3.7.1 plugin (https://cytoscape.org/) (21) was utilized to construct a PPI network of the interaction between DEGs with criteria were confidence score >0.9. And the top 15 genes were identified as the hub genes by Maximal Clique Centrality (MCC) algorithm (22). Furthermore, we also analyzed the functions and networks of these hub genes through GeneMANIA (http://genemania.org) (23).

### 2.4 Function enrichment analysis on decidua-DEGs, placenta-DEGs and Mφ polarization DEGs

To preliminary explore the regulation effects of decidua-DEGs and placenta-DEGs on Mφ, we performed immune enrichment analysis on decidua-DEGs and placenta-DEGs by a Cytoscape plugin “ClueGO” under the criterion of *P*<0.05. ClueGO can classify non-redundant Gene Ontology (GO) terms and visualize the functionally related genes in a clustered network (24). Besides, to comprehend the biological processes that Mφ polarization DEGs were involved in, we performed Kyoto Encyclopedia of Genes and Genomes (KEGG), rectome and Wiki pathway enrichment analysis on the Webgestalt website (http://www.webgestalt.org/) (25) under the criterion of *P*<0.05.

### 2.5 Construction of co-expression network and hub module identification by WGCNA

The WGCNA is widely used to uncover critically interacted genetic modules and essential genes by linking gene networks to clinical traits. In this study, the WGCNA co-expression system was established to investigate the modules that were highly associated with URSA by using the “WGCNA” package in R software based on genes related to Mφ polarization. First, genes with variation higher than 25% across samples in the combined dataset were selected as the input data set for the subsequent WGCNA. Then, the outlier cases were removed by hierarchical clustering analysis with the “goodSamplesGenes” function. After that, the appropriate soft threshold was determined by using the pickSoftThreshold function and validated by the correlation between k and p (k). Subsequently, the correlation matrix was converted into an adjacency matrix, which was further processed into a topological overlap matrix (TOM). The dynamic tree cutting approach was performed to identify various modules. The relationship between these modules and URSA was investigated. Finally, a module with the greatest Pearson correlation coefficient was picked for further investigation.

### 2.6 Screening secreted protein-encoding DEGs from total DEGs

Secreted proteins are potential signaling molecules for intercellular communication. The DSCs-derived and trophoblast-derived secreted proteins may be the possible active molecules for regulating Mφ polarization in the maternal-fetal interface. And therefore, we focused on secreted protein-encoding genes in DSC derived-DEGs and trophoblasts derived-DEGs in URSA. In this part, we retrieved the Human Protein Atlas website (https://www.proteinatlas.org/) to download the dataset of secreted protein-encoding genes. And then, the overlapped genes of secreted protein-encoding genes and decidua-DEGs, placenta-DEGs and Mφ polarization DEGs were separately obtained by using the Venn diagram tool.

### 2.7 PPI analysis, hub gene identification and physicochemical property analysis on secreted protein-encoding DEGs

First, PPI analysis and hub genes screening based on the secreted protein-encoding DEGs were performed as mentioned above. The top 10 under the MCC algorithm were defined as hub secreted protein-encoding genes. Then, to further unravel the physicochemical properties of hub secreted protein-encoding genes, we acquired their multiple physicochemical property parameters through the online tool ProtParam website (https://web.expasy.org/protparam/, accessed on 06/13/2022) (26), including molecular weight, theoretical pI, extinction coefficient, instability index, lipid index, and hydrophilic grand average (GRAVY).

### 2.8 Function enrichment analysis

Besides, to annotate the functions of genes in hub module identification by WGCNA as well as secreted protein-encoding genes, we conducted GO and KEGG pathway analysis by Webgestalt. GO was extensively applied to comprehensively describe the properties of genes and gene products in organisms, including biological process (BP), cell components (CC), and molecular function (MF). KEGG is available for systematic analysis of gene functions, link genome information and functions, and is able to build modules through computer processing of biological processes. In the above analysis, the species was selected as “Homo sapiens”, and the reference set was selected as genome protein-coding. GO terms and pathways with a threshold of *P* value <0.05 were filtered, and the top 10 terms (with the highest degree values) were displayed in a bubble plot by the “ggplot2” package in R software.

### 2.9 Diagnostic value of the secreted protein-encoding genes for URSA

Machine learning provides optimal methods to improve the accuracy of diagnosis models. In this study, the least absolute shrinkage and selection operator (LASSO) regression analysis and support vector machine recursive feature elimination (SVM-RFE) were performed to identify secreted protein-encoding genes with diagnostic value, namely signature genes. LASSO regression analysis is a refined model by constructing a penalty function based on a linear regression model, which can improve the stability of the model and the classification efficacy. The “glmne” package in R software was utilized for LASSO regression analysis (27). Besides, SVM-RFE is a support vector machine based on an optimal feature selection algorithm that ranks features on the basis of a recursive feature deletion sequence (28). R package “e1071” was utilized to construct the SVM-RFE classifier and analyze the candidate biomarkers in RSA. What’s more, to evaluate the diagnostic value of these genes, receiver operating characteristic (ROC) curve was applied, and the area under the curve (AUC) was calculated *via* the pROC R package. In addition, “rms” package in R software was also utilized to establish the nomogram prediction model. Each secreted protein-encoding gene with diagnostic value was converted into an assessment point system in the model. And the total score determined the final risk assessment value. Besides, the performance of the nomogram was assessed by calibration and discrimination, which was respectively evaluated by a visual calibration plot and ROC through the “rms” and “pROC” packages in R software.

### 2.10 Validation of hub genes and signature genes in clinical samples

Decidua and villus tissues were collected from healthy women required for induced abortion for unwanted pregnancies and patients with URSA in Hangzhou Hospital of Traditional Chinese Medicine Affiliated to Zhejiang Chinese Medical University from December 2022 to March 2023. Women with the following criteria were excluded from the study: (a) symptoms of endocrine or metabolic disease, (b) abnormal karyotype analysis, (c) uterine abnormalities or other identifiable causes of miscarriage.

For the validation of hub genes, total RNA from decidua and villus tissues were extracted by Trizol method, reverse transcription was performed by HiFiScript cDNA Synthesis Kit (CW2569M; CWBIO), and real time PCR was performed by SYBR Green Pro Taq HS premixed qPCR kit (AG11701; ACCURATE BIOLOGY). Finally, two-step PCR reaction procedure was used to perform the reaction, and then the computed CT values were calculated by 2−(ΔΔCT) method. The primer sequences used for quantitative real-time PCR (qRT-PCR) were as follows:

MX1: Forward primer (5′-3′) CGGTTCTGGGTCGGAGGCTAC, Reverse primer (5′-3′) CTGG ATGGCGGCGTTCTTCAC; IFI27: Forward primer (5′-3′) TCACTGGGAGCAACTGGACTC TC, Reverse primer (5′-3′) TCGCAATGACAGCCGCAATGG; IFIT3: Forward primer (5′-3′) GGAGAATGGCGTGAACCTGGAAG, Reverse primer (5′-3′) TTGAGATGGAGTCTTGCTC TGTTGC; LYN: Forward primer (5′-3′) TGAAGAGCGATGAAGGTGGCAAAG, Reverse primer (5′-3′) GTGACTCGGAGACCAGAACATTAGC; NNMT: Forward primer (5′-3′)AAG GGCTGAACTGATGGAAGGAATG, Reverse primer (5′-3′) CACTTCTGTACCACTGGAGC ACTG; SNCA: Forward primer (5′-3′) GTGGCAACAGTGGCTGAGAAGAC, Reverse primer (5′-3′) TACTGCTGTCACACCCGTCACC; IFIT1: Forward primer (5′-3′) CGAGAGCAGCCTT GGCAATGG, Reverse primer (5′-3′) TCCAGCAGTCCACTCACCTCAG; OAS2: Forward primer (5′-3′) CCAAAGAAGCGGGTGCCAGAC, Reverse primer (5′-3′) GAGAGCGAGTCC AGGGTAGAAGG.

As for the validation of signature genes decidua tissues were fixed with 4% paraformaldehyde and embedded in paraffin for immunofluorescence analysis, which was conducted following previously described methods (29). In this study, Vimentin representative decidual stromal cell and CX3CR1 representative macrophage. Digital pathological section (fluorescence) scanning analyzer (VS120-S6-W, OLYMPUS) was used to observe and photograph the images. Pearson correlation coefficient of FGF9-Vimentin, IL1R2-Vimentin, NID2-Vimentin, CFB-CX3CR1, CXCL11-CX3CR1 and NID2-CX3CR1 were performed using Image J software (version 1.53v). All samples were collected with the informed consent of patients, and all relevant procedures were approved by the Internal review and Ethics Committee of Hangzhou Hospital of Traditional Chinese Medicine affiliated to Zhejiang Chinese Medical University (NO. 2022LH007).

## 3 Results

### 3.1 Differential expression analysis to screen decidua-DEGs, placenta-DEGs, and Mφ polarization DEGs of URSA

**Figure 2:**
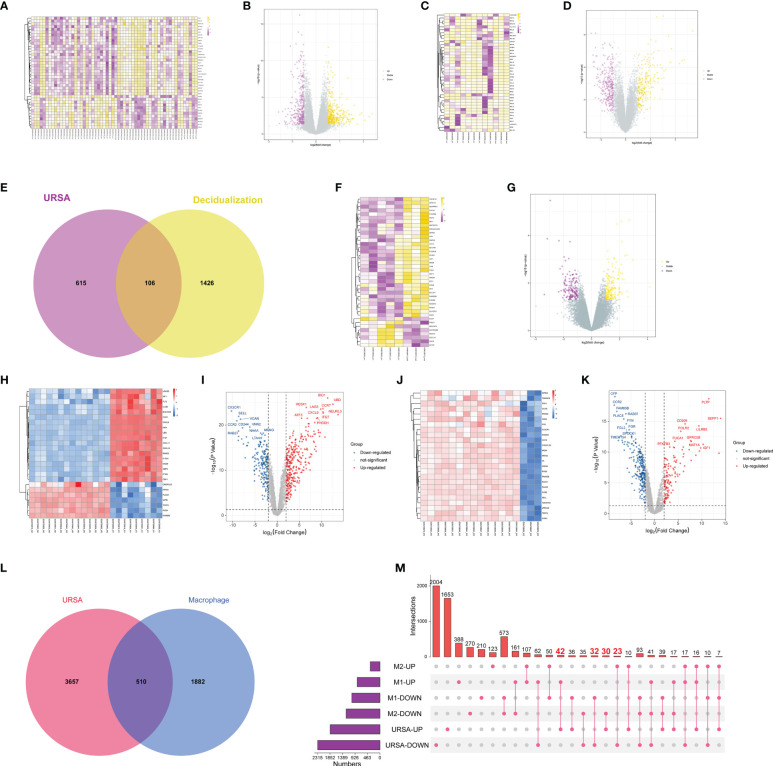
Differential expression analysis on genes at maternal-fetal interface of URSA. **(A)** Heat map and **(B)** volcano plot visualized the decidua-DEGs of URSA. **(C)** Heat map and **(D)** volcano plot visualized the decidualization related genes. **(E)** Venn diagram showed DSCs-DEGs by intersecting decidua-DEGs and decidualization related genes. **(F)** Heat map and **(G)** volcano plot visualized the placenta-DEGs of URSA. **(H)** Heat map and **(I)** volcano plot visualized the DEGs screened from the M0 and M1 macrophages. **(J)** Heat map and **(K)** volcano plot visualized the DEGs screened from the M0 and M2 macrophages. Venn diagram **(L)** and upset diagram **(M)** showed the intersection of decidua-DEGs with macrophage polarization related genes and decidua-DEGs, namely macrophage polarization DEGs. In the heatmap, each row represented a DEG, each column represents a sample, and in the volcano plot, each dot represents a gene, in which yellow and red plot points represented upregulated genes, and purple plot and blue points represent down-regulated genes.

After preprocessing and normalization of the included datasets, the limma package in R software was utilized to find the DEGs of URSA in decidual tissue (decidua-DEGs) as well as in placenta tissue (placenta-DEGs), with the screening conditions of *P* value <0.05 and |log2FC|>0.5. A total of 721 decidua-DEGs and 613 placenta-DEGs were obtained. Besides, 1532 genes related to induced decidualization derived from GSE94644 were intersected with decidua-DEGs, and 106 overlapped genes were acquired, which were taken as DEGs of DSCs in URSA, namely DSCs-DEGs. The DEGs mentioned above were separately visualized using a heatmap and volcano map, as shown in **Figures 2A-G**.

Furthermore, we obtained 1901 M1 polarization-related DEGs and 1622 M2 polarization- related DEGs from the GSE30595 data set with the same filter conditions as mentioned above. The heat map and volcano map were shown in **Figures 2H-L**. And there were 510 overlapped genes after intersecting the Mφ polarization-related DEGs with decidua-DEGs, namely Mφ polarization DEGs. And then, the upset diagram was plotted based on M1 and M2 polarization-related genes with decidua-DEGs, and the results manifested that 42 genes promoting M1 polarization were upregulated in URSA and 32 genes inhibiting M1 polarization were down-regulated in URSA. Besides, 23 genes promoting M2 polarization were downregulated in URSA, and 30 genes suppressing M2 polarization were up-regulated in URSA (**Figure 2M**).

### 3.2 Construction of PPI network and identification of hub genes

**Figure 3:**
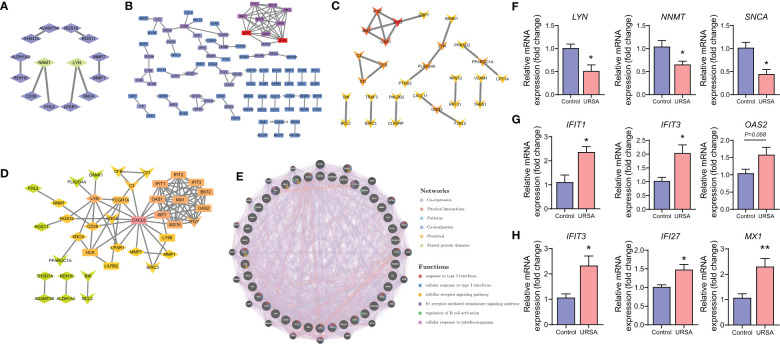
Protein-protein interaction analysis on DEGs at maternal-fetal interface. PPI network of DSC-DEGs **(A)**, placenta-DEGs **(B)** and macrophages polarization DEGs **(C)**. The PPI network of hub genes **(D)**, in which the diamonds, the rectangles and the “V”-shape nodes represented hub genes from DSC-DEGs, from macrophages polarization DEGs, and from placenta-DEGs, respectively. The functions of the hub proteins were predicted by the GeneMANIA plug-in in Cytoscape **(E)**. The qRT-PCR results of DSC-DEGs **(F)**, placenta-DEGs **(G)** and macrophages polarization DEGs **(H)**. * represents *P*<0.05, **represents *P*<0.01, compared with the control group.

Proteins are the primary carriers of biological activity. To understand how proteins encoded by these DEGs interact, DSCs-DEGs, placenta-DEGs, and Mφ polarization DEGs were uploaded to the STRING website to construct the PPI network, respectively, as shown in **Figures 3A-C**. Subsequently, hub genes in networks were detected by the MCC algorithm (Cytoscape, CytoHubba) (30). Hub genes in DSCs included NNMT, LYN, SNCA. Hub genes derived from the placenta contained IFIT1, IFIT3, OAS2, etc. Decidual Mφ derived hub genes involved IFIT3, IFI27, MX1, etc. (**Table 2**). After that, the hub genes were converged for PPI analysis, which exhibited close interactions between these hub genes (**Figure 3D**). Furthermore, we also analyzed the functions and networks of these hub genes through GeneMANIA, and the result suggested that these genes were closely linked both in localization and functions and were mainly involved in the regulation of immune activity, including response to type I interferon, toll-like receptor signaling pathway, regulation of B cell activation and so on (**Figure 3E**). Finally, we verified the obtained hub genes with clinical samples by qRT-PCR. The results showed that for DSCs-hub DEGs, LYN, NNMT and SNCA significantly decreased in URSA group (*P*<0.05) (**Figure 3F**), for placenta-hub DEGs, IFIT1 and IFIT3 significantly increased in URSA group (*P*<0.05) (**Figure 3G**), while OAS2 tends to be elevated in URSA (*P*=0.058). As for Mφ polarization associated hub DEGs, IFIT3, IFI27 and MX1 were significantly increased in URSA group (for IFIT3 and IFI27, *P*<0.05; for MX1, *P*<0.01) (**Figure 3H**).

**Table 2:** Identification of Hub Genes.

Gene name	MCC	Source	Gene name	MCC	Source
LYN	2	URSA DSCs*	CFI	2	URSA macrophage
NNMT	2	URSA DSCs	C3	2	URSA macrophage
SNCA	1	URSA DSCs	LYN	2	URSA macrophage
RGS16	1	URSA DSCs	VCAM1	1	URSA macrophage
RGS11	1	URSA DSCs	THBS1	1	URSA macrophage
MMP7	1	URSA DSCs	RPS12	1	URSA macrophage
MMP1	1	URSA DSCs	RPL31	1	URSA macrophage
LPAR1	1	URSA DSCs	IFIT1	40322	URSA placenta
FHL2	1	URSA DSCs	IFIT3	40322	URSA placenta
CD38	1	URSA DSCs	MX1	40320	URSA placenta
RDH10	1	URSA DSCs	ISG15	40320	URSA placenta
ALDH1A2	1	URSA DSCs	OAS1	40320	URSA placenta
THSD7A	1	URSA DSCs	IRF7	40320	URSA placenta
ADAMTS8	1	URSA DSCs	OAS2	40320	URSA placenta
IFIT3	7	URSA macrophage	IFIT2	40320	URSA placenta
IFI27	6	URSA macrophage	BST2	40320	URSA placenta
MX1	6	URSA macrophage	HCK	5	URSA placenta
OAS1	6	URSA macrophage	FCGR1A	3	URSA placenta
PLA2G4A	2	URSA macrophage	CXCL8	3	URSA placenta
GNG2	2	URSA macrophage	TLL2	3	URSA placenta
PPARGC1A	2	URSA macrophage	LY96	3	URSA placenta
CFB	2	URSA macrophage	LILRB2	3	URSA placenta

*There only 14 hub genes in URSA DSCs.

### 3.3 Immunoenrichment analysis on decidua-DEGs and placenta-DEGs in URSA

**Figure 4:**
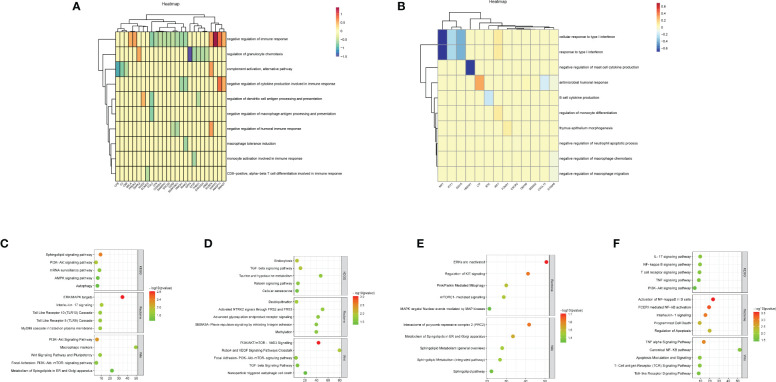
Functional analysis of DEGs to reveal cell-cell interactions at maternal-fetal interface The heatmap visualized immune enrichment analysis of decidua-DEGs **(A)** and placenta-DEGs **(B)** by ClueGO plug-in in Cytoscape. The bubble chart displayed the functional enrichment analysis of the upregulated genes that promoted M1 polarization in URSA **(C)**, the downregulated genes in URSA that inhibited M1 polarization **(D)**, the downregulated genes that promoted M2 polarization **(E)** and the upregulated genes that inhibited M2 polarization **(F)**.

We performed immune enrichment analysis on decidua-DEGs and placenta-DEGs using the “ClueGO” plugin in Cytoscape. The results demonstrated that decidua-DEGs in URSA were mainly enriched in negative regulation of immune response, Mφ tolerance induction, and negative regulation of Mφ antigen processing and presentation. And placenta-DEGs in URSA were mainly enriched in the cellular response to type I interferon, Mφ tolerance induction, regulation of Mφ antigen processing and presentation, Mφ activation, and so on (**Figures 4A, B**). From the above analysis, we could indicate that DEGs in the decidua and placenta of URSA were involved in various immune processes, and the regulation effects on Mφ were one of its important manifestations.

### 3.4 Functional enrichment analysis of Mφ polarization DEGs in URSA

To comprehend the biological processes that are associated with Mφ polarization DEGs, we performed KEGG, rectome and Wiki pathway enrichment analysis by using Webgestalt. The results indicated that the upregulated genes that promoted M1 polarization in URSA were mainly enriched in Sphingolipid signaling pathway, ERK/MAPK signaling pathway, interleukin-17 signaling pathway, PI3K-Akt signaling pathway, etc.(**Figure 4C**). The downregulated genes in URSA that inhibited M1 polarization were associated with TGF-β signaling pathway, cellular senescence, deubiquitination, PI3K/AKT/mTOR-VitD3 signaling pathway and TGF-β receptor signaling, etc.(**Figure 4D**) The downregulated genes that promoted M2 polarization were primarily involved in Pink/Parkin mediated mitophagy, mTORC1 mediated signaling pathway, metabolism of Spingolipids in ER and Golgi apparatus, etc.(**Figure 4E**). The upregulated genes that inhibited M2 polarization in URSA were enriched in VEGF signaling pathway, apoptosis, activation of NF-kappaB in B cells, and TNF alpha signaling pathway (**Figure 4F**). From the above results, it can be inferred that DEGs that tend to enhance the M1/M2 ratio in URSA could activate pro-inflammatory responses and regulate intercellular communication and cell fate, etc.

### 3.5 WGCNA for Mφ polarization related genes and enrichment analysis on genes in critical modules

**Figure 5:**
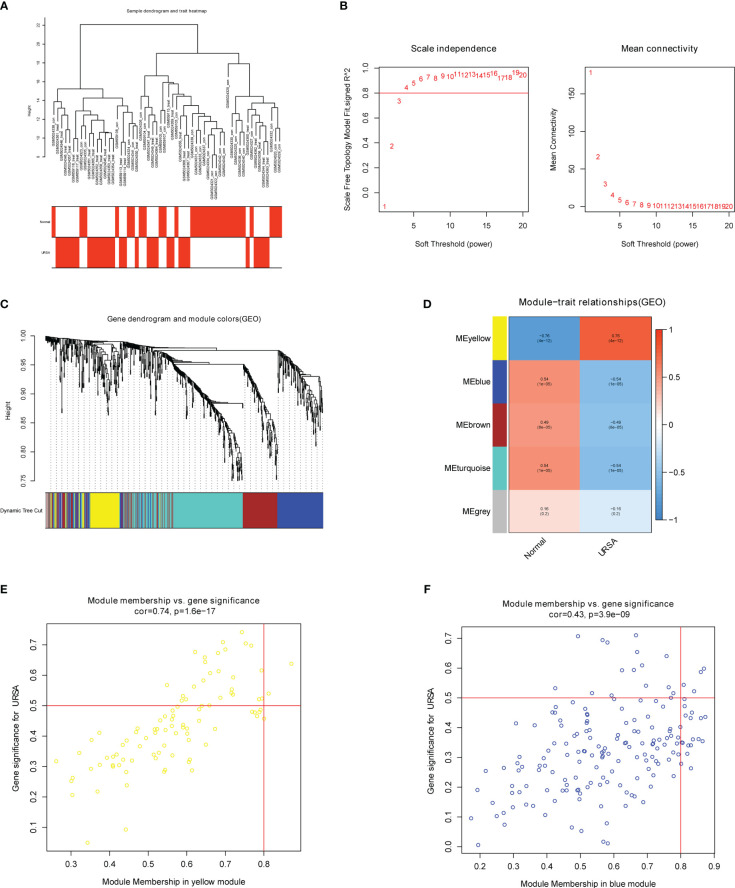
WGCNA for macrophage polarization related genes **(A)** Clustering dendrogram of samples with trait heatmap. **(B)** Analysis of the scale-free fit index and the mean connectivity for various soft-thresholding powers. **(C)** Clustering dendrogram of genes by average linkage hierarchical clustering, in which 4 modules were identified. Each branch represents a gene, and each color underneath the branch represents a co-expression module. **(D)** Heat map visualizing the correlation between modules and RPL. **(E, F)** A scatter plot showing the correlationship between MM (X-axis) vs. GS (Y-axis) in the yellow and blue modules.

The co-expression network was conducted with WGCNA package to detect the critically interacted genetic modules based on the mRNA expression profiling of Mφ polarization- related genes. After matching the sample traits with the expression matrix, the sample cluster tree was shown in **Figure 5A**. The power of β= five was selected as the soft thresholding power to ensure a scale-free network according to the scale independence and mean connectivity values (**Figure 5B**). In total, four modules were identified by WGCNA (**Figure 5C**). The specific module information was shown in **Table 3**. Then heatmaps of module-trait relationships were plotted to assess the correlation between each module and clinical traits (URSA and health control), as shown in **Figure 5D**. Besides, **Figure 5E** displayed that genes in the yellow module were significantly positively correlated with URSA modules (cor=0.76, *P*=4e−12), and genes in the blue module were significantly negatively correlated with URSA (cor=-0.54, *P*=1e−05) (**Figure 5F**). Considering that the blue and yellow modules were the most significantly related to URSA among all the modules, genes in the two modules were picked for further functional enrichment analysis.

**Table 3:** Module characteristics within each module.

Module	Expression in URSA	No. genes in module
blue	DOWN	173
brown	DOWN	158
turquoise	DOWN	292
yellow	UP	95

**Figure 6:**
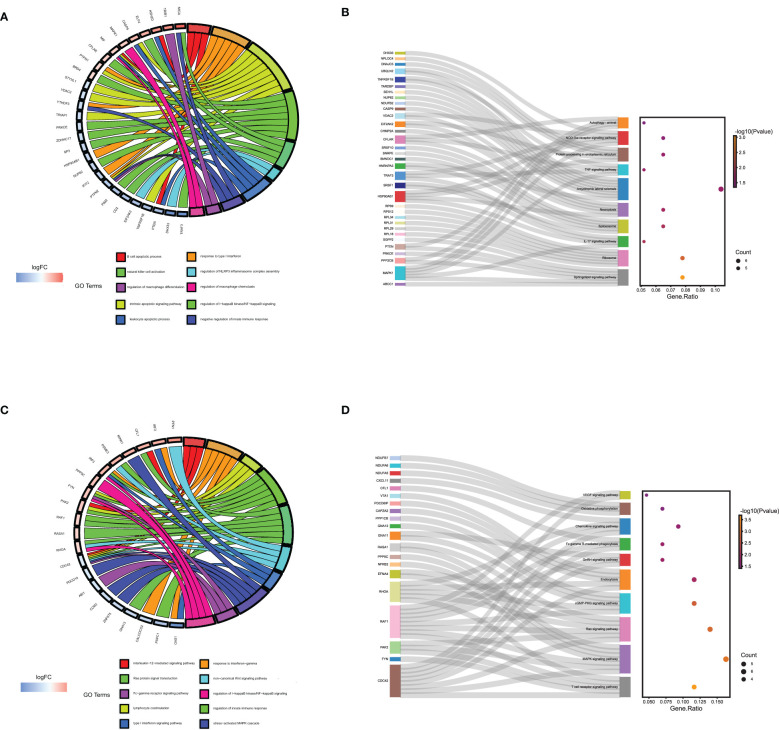
The GO and KEGG enrichment analysis of genes in the yellow and blue module derived from WGNCA. The GO **(A)** and KEGG enrichment analysis **(B)** of genes in the yellow module by Webgestalt. The GO **(C)** and KEGG enrichment analysis **(D)** of genes in the blue module by Webgestalt.

The BP of GO enrichment analysis illustrated that genes in the blue module were enriched in negative regulation of the immune system process, regulation of Mφ differentiation and Mφ chemotaxis, etc. In KEGG analysis, the enriched pathways of blue module genes were necroptosis, IL-17 signaling pathway, TNF signaling pathway, and autophagy, etc. (**Figure 6A, B**) Genes in yellow modules are mainly involved in the BP of positive regulation of leukocyte proliferation, activating cell surface receptor signaling pathway, and Ras protein signal transduction, etc. As for KEGG pathway analysis, genes in yellow modules were enriched in T cell receptor signaling pathway, MAPK signaling pathway, Fc gamma R-mediated phagocytosis, and chemokine signaling pathway, etc. (**Figures 6C, D**) From the above results, it could be assumed that the blue module genes mainly functioned positively for embryonic immune tolerance, while the yellow module genes were chiefly involved in embryonic immune rejection. In addition, we also discovered that Mφ polarization DEGs communicated intracellular and extracellular signaling cascades to achieve information exchange between cells. However, the specific communication mechanism remained to be further explored.

### 3.6 Identification of the secreted protein-encoding genes at the maternal-fetal interface in URSA

**Figure 7:**
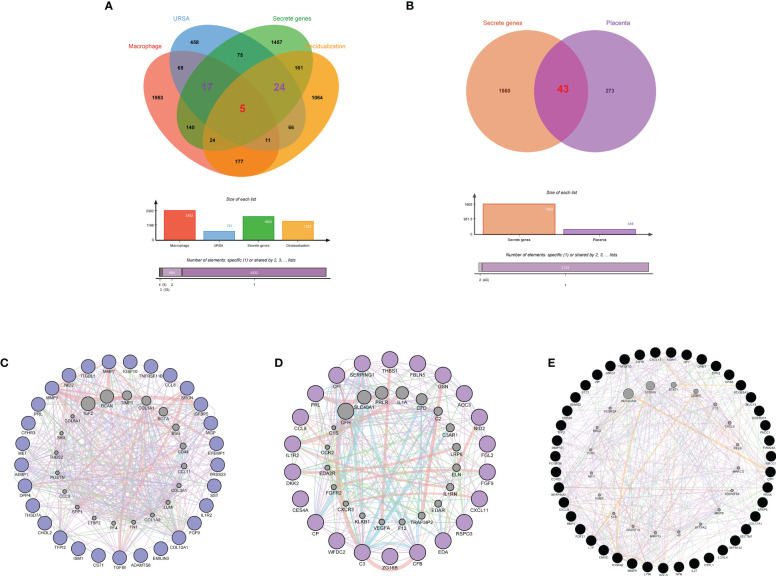
Identification of secreted protein encoding genes at maternal fetal interface in URSA and PPI analysis on them. Venn diagram of secreted protein encoding gene sets, decidua-DEGs, decidualization related genes and macrophages polarization related genes **(A)**. Venn diagram of secreted protein encoding gene sets and placenta-DSCs **(B)**. PPI network of secreted protein encoding DEGs derived from DSCs **(C)**, macrophages **(D)** and placenta **(E)**.

Secreted proteins are pivotal mediators of intercellular communication. To clarify the intercellular communication at the maternal-fetal interface in RSA, we identified DEGs that could encode secreted proteins from DSCs, placenta, and decidual Mφ, separately. Firstly, a total of 1903 secreted were obtained from the Human Protein Atlas website. Subsequently, the decidua-DEGs, along with the decidualization-related DEGs were intersected with the 1903 secreted proteins, and a total of 29 secreted protein-encoding DEGs of DSCs in URSA were obtained. In addition, we intersected decidua-DEGs, Mφ polarization related genes in the 1903 secreted proteins, and finally obtained 22 decidual Mφ derived secreted proteins encoding DEGs (**Figure 7A**). Similarly, 43 secreted protein encoding DEGs derived from the placenta were obtained by intersecting placenta DEGs with the 1903 secreted proteins (**Figure 7B**).

### 3.7 PPI network construction, hub gene identification, and physicochemical property analysis of secreted proteins

PPI networks were constructed for **s**ecreted proteins encoding genes from DSCs, placenta and decidual Mφ by GeneMANIA, and hub-secreted proteins encoding genes were screened by MCC algorithm in cytoHubba of Cytoscape software. In DSCs, the hub-**s**ecreted proteins encoding genes included MMP1, COL12A1, MET, MMP7, and TNFRSF11B, etc. (**Figure 7C**). The hub-secreted proteins encoding genes of decidual Mφ covered C3, CFB, SERPING1,CP and CFI (**Figure 7D**). In the placenta, the hub-**s**ecreted protein-encoding genes consist of CXCL8, SERPINA1, FCGR3B, S100A8, and LTF, etc. (**Figure 7E**). Besides, we reported the physicochemical properties of the identified hub-secreted proteins, which is vital for the in-depth investigation of the significant biomolecules. The detailed information about hub-secreted protein-encoding genes was shown in **Table 4**.

**Table 4:** The physicochemical properties of the reported hub proteins.

Name	Number of Amino Acids	Molecular Weight (kda)	Theoretical pI	Number of Negatively Charged Residues(Asp + Glu)	Number of Positively Charged Residues(Arg + Lys)	Extinction Coefficient	Instability Index	Aliphatic Index	Grand Average of Hydropathicity (GRAVY)	Classification
MMP1	469	54006.90	6.47	58	54	76905	35.46	65.27	-0.572	DSCs
COL12A1	3063	333146.77	5.38	366	313	334620	32.90	75.45	-0.427	DSCs
MET	1390	155541.31	7.02	137	134	127070	42.61	88.02	-0.144	DSCs
MMP7	267	29676.84	7.73	28	29	44015	32.39	76.37	-0.369	DSCs
TNFRSF11B	401	46026.02	8.66	42	53	50890	49.09	74.84	-0.483	DSCs
C3	1663	187148.06	6.02	213	195	180055	42.47	87.87	-0.320	Decidua macrophage
CFB	764	85532.87	6.67	94	91	122170	43.55	75.09	-0.501	Decidua macrophage
SERPING1	500	55154.19	6.09	48	43	27180	33.71	89.70	-0.125	Decidua macrophage
CP	3785	311348.49	4.82	0	0	47000	48.21	32.66	0.817	Decidua macrophage
CFI	583	65750.26	7.72	67	70	101405	38.53	70.53	-0.321	Decidua macrophage
CXCL8	99	11098.12	9.10	11	16	7240	24.95	101.52	-0.019	Placenta
SERPINA1	418	46736.55	5.37	56	41	25565	31.65	91.20	-0.183	Placenta
FCGR3B	233	26215.86	6.22	21	18	46660	40.91	90.73	-0.080	Placenta
S100A8	93	10834.51	6.50	15	14	11460	25.04	96.45	-0.397	Placenta
LTF	710	78181.95	8.50	79	90	88290	44.28	74.62	-0.337	Placenta

*Extinction coefficients are in units of M^-1^ cm^-1^ , at 280 nm measured in water.

### 3.8 Functional enrichment analysis of secreted proteins encoding DEGs at maternal-fetal interface in URSA

**Figure 8:**
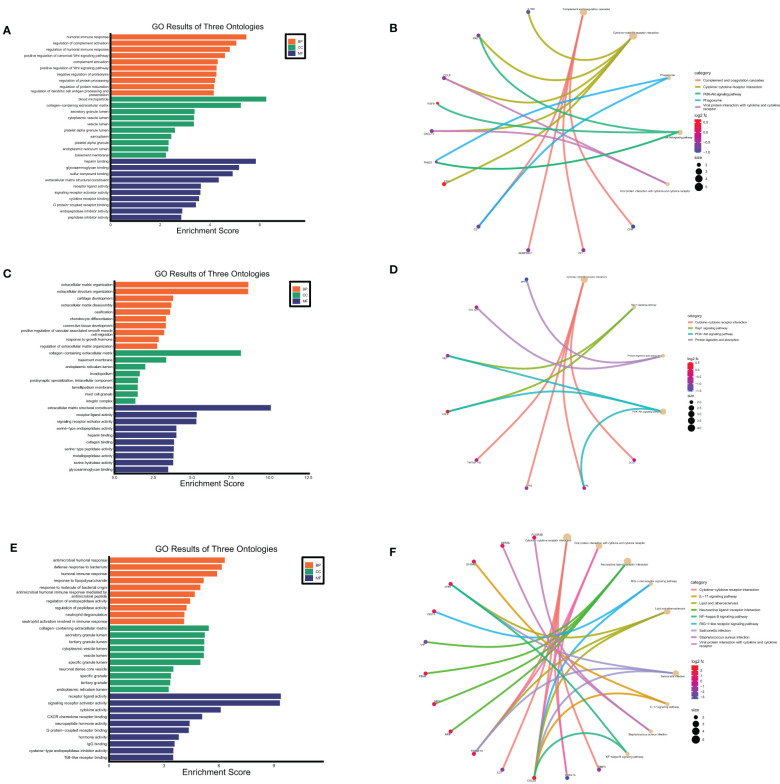
Functional enrichment analysis of secreted proteins encoding DEGs at maternal fetal interface in URSA. The barplot showed the results of GO enrichment analysis on secreted protein encoding DEGs derived from DSCs **(A)**, macrophages **(C)** and placenta **(E)**. The circular network plot visualized the results of KEGG analysis on secreted protein encoding DEGs derived from DSCs **(B)**, macrophages **(D)** and placenta **(F)**, in which khaki nodes represented terms, the color from blue to red nodes represented secreted protein encoding DEGs, and connecting lines represented the relationship between terms and genes.

GO and KEGG enrichment analysis of **s**ecreted proteins encoding DEGs of DSCs, placental and decidual Mφ was respectively performed in this analysis. The 29 secreted proteins encoding DEGs of DSCs were mainly distributed in the cellular component of collagen-containing extracellular matrix, basement membrane, integrin complex, etc., with molecular functions such as receptor-ligand activity, integrin binding and transmembrane receptor protein kinase activity. These genes were predominantly involved in BPs of extracellular matrix organization, positive regulation of protein kinase B signaling, and female pregnancy (**Figure 8A**). KEGG enrichment analysis corroborated that these 29 DEGs were mainly enriched in cytokine-cytokine receptor interaction, PI3K-Akt signaling pathway, Rap1 signaling pathway, and so on (**Figure 8B**).

GO enrichment analysis on the 22 secreted-protein encoding DEGs from decidual Mφ implied that these genes were primarily expressed in collagen-containing extracellular matrix, secretory granule lumen and cytoplasmic vesicle lumen, mainly affected receptor ligand activity, cytokine receptor binding and chemokine activity, and involved in humoral immune response, regulation of complement activation, regulation of dendritic cell antigen processing and presentation, etc.(**Figure 8C**). KEGG analysis on these genes suggested that these genes primarily participated in cytokine-cytokine receptor interaction, PI3K-Akt signaling pathway and positive regulation of Wnt signaling pathway (**Figure 8D**).

As for the 43 secreted protein encoding DEGs from placenta of URSA, the results of GO-CC analysis verified that DEGs were mainly enriched in collagen-containing extracellular matrix, cytoplasmic vesicle lumen, secretory granule lumen. And GO-BP analysis indicated that DEGs were mainly involved in humoral immune response, response to lipopolysaccharide and neutrophil degranulation, etc. Regarding GO-MF analysis, the 43 DEGs were primarily related to cytokine activity, CXCR chemokine receptor binding and IgG binding, etc.(**Figure 8E**). The KEGG analysis indicated that the 43 DEGs mainly participated in cytokine-cytokine receptor interaction, IL-17 signaling pathway and NF-kappa B signaling pathway (**Figure 8F**).

### 3.9 Correlations between cells at the maternal-fetal interface evaluated by hub secreted protein-encoding genes of donor cells and hub genes in recipient cells

To investigate the cellular communication between DSC, placenta, and Mφ in URSA, we applied Spearman analysis test to detect the correlation between DSC or placenta-derived hub secreted protein-encoding genes and the hub genes of Mφ, along with the correlation between Mφ derived critical secreted proteins and the hub genes of DSC or placenta in URSA samples.

**Figure 9:**
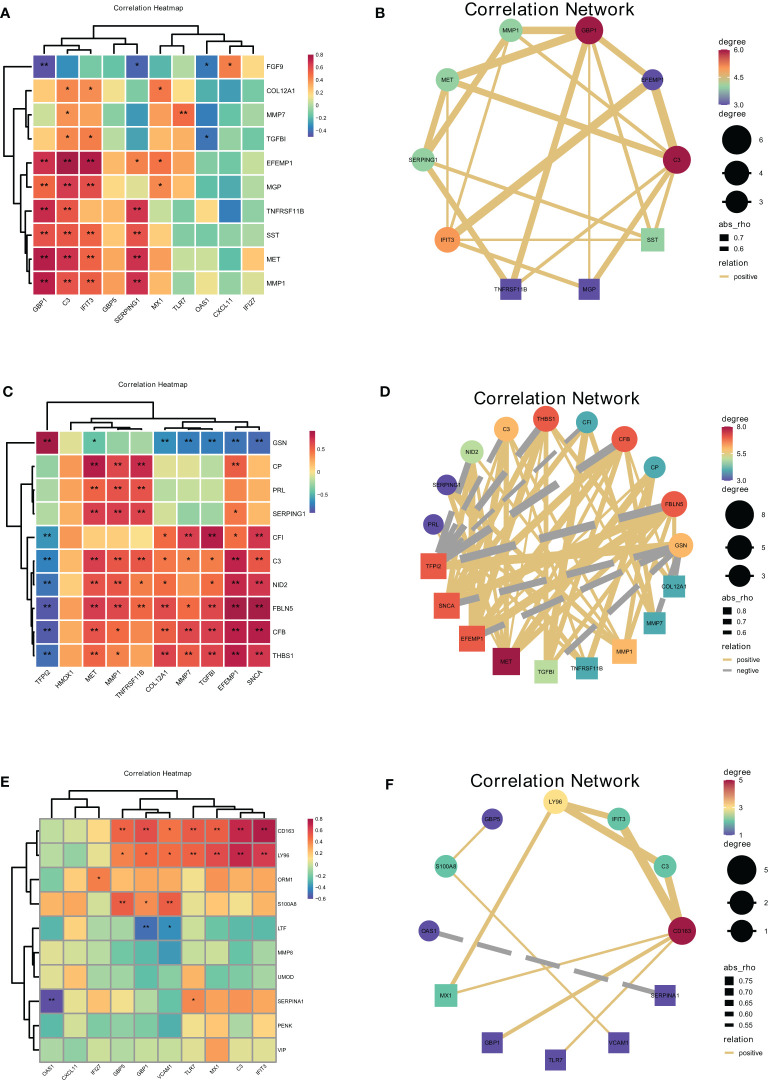
Correlations between cells at the maternal-fetal interface evaluated by hub secreted protein encoding genes of donor cells and hub genes in recipient cells. The heatmap and correlation network visualized the correlation of DSCs-derived secreted encoding DEGs with macrophage polarization DEGs **(A, B)**, macrophage-derived secreted encoding DEGs with DSC-DEGs **(C, D)**, placenta-derived secreted encoding DEGs with macrophage polarization DEGs **(E, F)**. *represents P<0.05, **represents P<0.01.

Spearman correlation analysis on hub secreted protein encoding genes of DSCs and hub genes of decidual Mφ demonstrated that EFEMP1 was closely and positively correlated with C3 (r=0.78, *P*<0.01), followed by EFEMP1 with IFIT3 (r=0.76, *P*<0.01) and MET with GBP1 (r=0.74, *P*<0.01). Besides, FGF9 was closely and negatively correlated with GBP1 (r=-0.48, *P*<0.01), followed by FGF9 with SERPING1 (r=-0.42, *P*<0.05), TGFBI with OAS1 (r=-0.38, *P*<0.05) (**Figures 9A, B**).

Furthermore, the correlation analysis between Mφ derived hub secreted-protein-encoding genes and hub genes of DSC confirmed that GSN was closely and positively correlated with TFPI2 (r=0.89, *P*<0.01), followed by FBLN5 with SNCA (r=0.88, *P*<0.01), FBLN5 with EFEMP1 (r=0.81, *P*<0.01), and FBLN5 with TFPI2 (r=-0.85, *P*<0.01). Besides, FBLN5 was closely negatively correlated with TFPI2 (r=-0.85, *P*<0.01), followed by CFB with TFPI2 (r=-0.84, *P*<0.01), and GSN with SNCA (r=-0.78, *P*<0.01). This result illustrated the close correlation between decidual Mφ and DSC. Heat maps and network plots were plotted to visualize gene correlations (**Figures 9C, D**).

The correlation analysis of placental derived hub secreted-protein-encoding genes and hub genes of decidual Mφ identified the highest positive correlation coefficient between CD163 and IFIT3 (r=0.75, *P*<0.01), followed by CD163 and C3, as well as LY96 and C3 (r=0.70, *P*<0.01). And SERPINA1 was the most negatively correlated with OAS1 (r=-0.61, *P*<0.01), followed by LTF with GBP1 (r=-0.50, *P*<0.01) (**Figures 9E, F**).

### 3.10 Identifying the diagnostic value of secreted proteins by machine learning and immunofluorescence analysis

**Figure 10:**
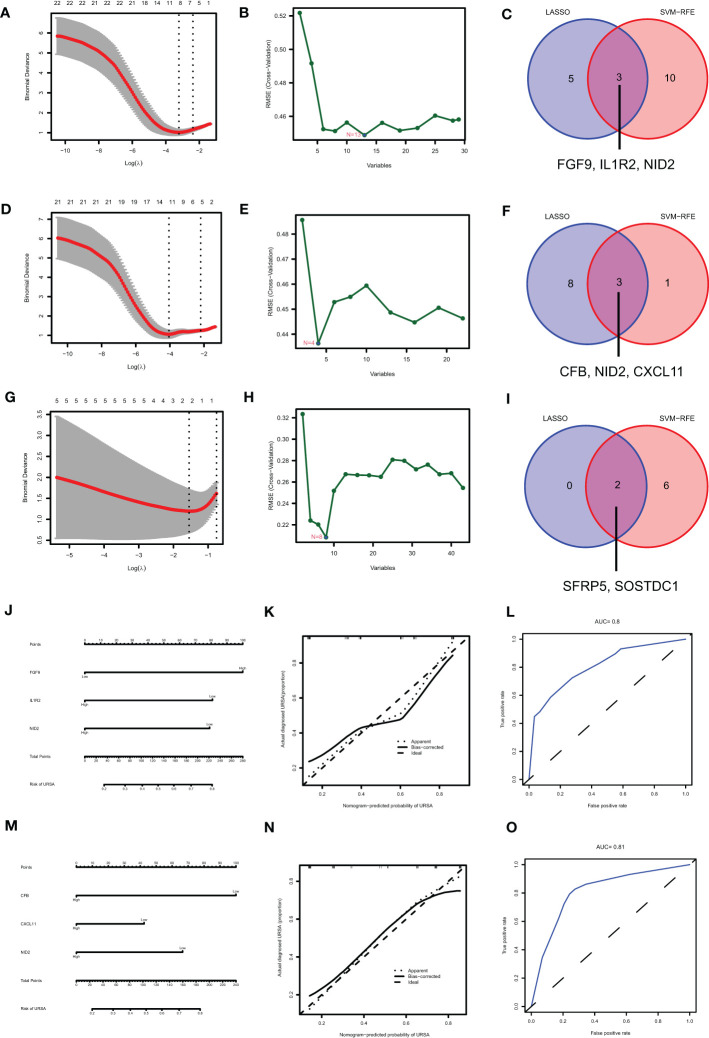
Identifying the diagnostic value of secreted proteins by machine learning. Results of LASSO, SVM-RFE algorithms and the intersection of the two for screening signature genes from DSC-DEGs **(A–C)**, macrophage polarization DEGs **(D–F)** and placenta-DEGs **(G–I)**. Nomograms of the 3 DSC-derived signature genes **(J)** and 3 decidual macrophage-derived genes **(M)**, and the corresponding calibration curves **(K, N)** and ROCs **(L, O)**.

**Figure 11:**
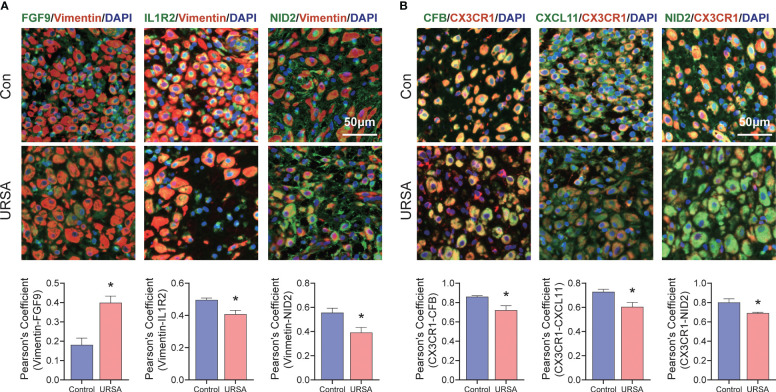
Validation of signature genes in clinical samples. **(A)** Immunofluorescence results of FGF9-Vimentin, IL1R2-Vimentin and NID2-Vimentin. **(B)** Immunofluorescence results of CFB-CX3CR1, CXCL11-CX3CR1 and NID2-CX3CR1. *represents *P*<0.05, compared with the control group; Here, Vimentin representative decidual stromal cell, CX3CR1 representative macrophage; Scale bar: 50μm.

Maternal-fetal interface-derived secreted proteins may be candidate biomarkers for the diagnosis of URSA. To determine their diagnostic value, LASSO and SVM-RFE algorithms were conducted and the ROC curves of each hub gene were plotted using R software. In DSCs, 8 genes and 13 genes were respectively obtained by LASSO and SVM-RFE, and there were three overlapped genes, including FGF9, IL1R2, NID2 (**Figures 10A–C**). Besides, we got 11 genes by LASSO regression and 4 genes by SVM-RFE from 22 secreted proteins encoding genes of decidual Mφ, the intersection of which contains three genes, including CFB, NID2, CXCL11 (**Figures 10D–F**). Similarly, we obtained 2 genes from 43 URSA placental secreted proteins by LASSO regression and 8 genes by SVM-RFE, with the intersecting genes being SFRP5 and SOSTDC1 (**Figures 10G–I**). The AUC of all these signature genes were above 0.65, indicating a high diagnostic accuracy (**Supplementary Figure 2**). In addition, we constructed nomogram with the three DSC-derived and three decidual Mφ-derived genes, respectively. And the calibration curves verified the predictive performance of the model, with high AUC value to verify its robustness (**Figures 10J–O**). Finally, we verified the obtained signature genes with decidual tissue by immunofluorescence colocalization analysis. The results showed that FGF9, IL1R2 and NID2 colocalized with the DSC marker vimentin. Furthermore, FGF9 were significantly increased in URSA (*P*<0.05), IL1R2 and NID2 were significantly decreased in URSA (*P*<0.05). Besides, CFB, CXCL11 and NID2 were colocalized with the Mφ marker CX3CR1, all of which were significantly decreased in URSA (*P*<0.05) (**Figure 11**).

## 4 Discussion

This study obtained 721 decidua-DEGs, 613 placenta-DEGs, and 510 Mφ polarization DEGs. Besides, hub genes were screened by CytoHubba and validated by qRT-PCR, including LYN, NNMT and SNCA in DSCs; IFIT1, IFIT3 and OAS2 in placenta; IFIT3, IFI27 and MX1 associated with Mφ polarization in URSA. Besides, functional analysis of the hub genes by GeneMANIA suggested that they were closely related to immune regulation. For example, LYN is an non-receptor tyrosine-protein kinase and is an essential regulator of immunoreceptor signaling, initiating both pro-inflammatory and suppressive signaling pathways in myeloid immune cells (e.g. macrophages, neutrophils, dendritic cells and monocytes) (31). Studies found that Toll-like Receptor (TLR) pathways, which trigger inflammatory responses to pathogen-associated molecular patterns (PAMPs) are also regulated by LYN. Downstream of TLRs, the adaptor proteins MyD88 and CARD9 and the transcription factor IRF5 generate hyperactivated signaling in LynKO myeloid cells and drive autoimmunity in LynKO mice. Furthermore, one study found a negative role for LYN in macrophage TLR4 signaling (32). Our study is the first to discover that LYN was decreased in DSCs of URSA, and it might be one of the molecular mechanisms that accounted for Mφ polarization disorder in URSA. Besides, for placental-derived IFIT1, IFIT3 (respectively encoding interferon induced protein with tetratricopeptide repeats 1 and 3) and OAS2 (encoding 2’-5’-Oligoadenylate Synthetase 2), along with IFIT3, IFIT27 and MX1 (encoding MX Dynamin Like GTPase 1) related to Mφ polarization, all these genes belong to the core responsive IFN-inducible genes. They are all closely associated with cytokine signaling in immune system, such as the pro-inflammatory phenotypic transformation of macrophages (33, 34). Currently, these genes are supposed to play an influential role in embryo implantation (35) and infection-related diseases during pregnancy (36). However, in the context of URSA, their expression patterns and biological significance have not been studied. Our results discovered that IFIT1, IFIT3, OAS2, IFIT27 and MX1 are elevated in URSA, which may represent the activation of immune and inflammatory responses in the maternal-fetal interface. This is consistent with the current findings (37). For instance, Wang et al. clarified that decidual stromal cell-derived decorin can polarize decidual macrophages toward the M1 phenotype and results in the occurrence of URSA (38). Gao et al. confirmed that the absence of G-CSF derived from trophoblasts weakened the suppression of trophoblasts against macrophages and may be a key factor in URSA occurrence (39). All these findings reveal that gene expression profile disorders in DSCs and trophoblast cells can disturb the immune landscape at the maternal-fetal interface, especially the communication with macrophages, which is an essential hint to explain the pathological mechanism of URSA. And our findings provide new targets and direction for research in this area.

To further systematically understand the immunomodulatory activities that decidua-DEGs and placenta-DEGs were involved in, we performed immune function enrichment analysis by CluGO, which displayed that these DEGs were mainly closely related to macrophage activity, such as affecting macrophage tolerance induction, regulation of macrophage antigen processing and presentation as well as macrophage activation. It has been confirmed that DSC-conditioned medium (CM) can promote macrophage survival and induce typical phenotype of decidual macrophages, and that DSC-CM also inhibit the pro-inflammatory effects of granulocyte-macrophage colony-stimulating factor (GM-CSF) by upregulating CD14, CD163 and CD209 (40). In addition, scholars have found that the level of nuclear factor-κB ligand (RANKL)/RANK levels at the maternal-fetal interface were abnormally decreased in patients with miscarriage, and RANKL receptor activator, secreted by trophoblasts and DSCs, could polarize dMϕ toward a M2 phenotype (41). Besides, Wu et al. demonstrated that overexpression of miR-410-5p in trophoblasts inhibited the polarization of M2 macrophages (42). Chen et al. discovered that reduced IL-10 derived from placental villi of URSA patients could activate endoplasmic reticulum stress of macrophages, thereby inhibiting M2 polarization (43). From the above studies we could project that DSCs and trophoblasts exerted significant effects on the phenotype and function of decidual macrophage. Combined with the results of differential expression analysis in this study, we hypothesized that DEGs at the maternal-fetal interface in URSA may affect macrophage polarization and be closely associated with pregnancy outcome in URSA.

To further understand the molecular mechanisms of intercellular communication that Mφ was involved in at the maternal-fetal interface, we performed enrichment analysis on Mφ polarization DEGs as well as the key module gene based on macrophage polarization-related genes by WGCNA. The results revealed that these genes regulated immune inflammatory signaling molecules and influenced cell function and fate, which might be an important way for macrophages to participate in intercellular communication at the maternal-fetal interface. Studies have confirmed that plenty of macrophages accumulated rapidly in the superficial stroma of endometrium during the period of “implantation window” period, suggesting that macrophages may involve in endometrial tolerance (44). For example, M2 macrophages are the primary source of decidual retinoic acid regulating regulated gap junction communication and promoting the differentiation of endometrial stromal cells (45). In addition, Sheng et al. uncovered that decidual macrophages could clear apoptotic DSCs through cytoplasmic action under the regulation of the IL-33/ST2 axis, the disorder of which was closely associated with URSA (46, 47). Hao et al. demonstrated that macrophages could affect nitric oxide levels and induce trophoblast apoptosis *via* protein l-arginine methyltransferase 3 (PRMT3)/asymmetrical dimethylarginine (ADMA), which was considered as a potential cause of recurrent miscarriage (48). According to the above studies, we could intuitively conclude that the regulation of DSCs and trophoblasts by decidual macrophages was essential for physiological pregnancy and that abnormalities in this dialogue mechanism are closely associated with pregnancy loss.

Considering that secreted proteins may play a central role in communicating intercellular signal transduction, we screened the genes encoding secreted proteins from DEGs of URSA. And finally, we obtained 29 secreted protein encoding DEGs in DSC-DEGs, 43 secreted protein encoding DEGs in placenta-DEGs, and 22 secreted proteins encoding DEGs in decidual macrophages. The functional enrichment analysis argued that these genes were mainly enriched in cytokine-cytokine receptor interaction, PI3K-Akt signaling pathway, NF-kappa B signaling pathway, and Wnt signaling pathway, etc. Taking the PI3K-Akt signaling pathway as an example, it is an intracellular signaling pathway that regulates metabolism, cell proliferation, survival, and growth in response to extracellular signals. Several studies have confirmed that the PI3K/Akt signaling pathway at the maternal-fetal interface is involved in the pathology of the URSA mechanism (49). Cui et al. suggested that the abnormal polarization of macrophages in patients with spontaneous abortion was related to the activation of the PI3K/Akt signaling pathway (50). Dai et al. observed that DSC could promote M2-like phenotype polarization by secreting growth arrest-specific factor 6 (rhGAS6) and activating the PI3K/Akt signaling pathway, and this mechanism was disrupted in patients with miscarriage (51). In addition, studies confirmed that G-CSF derived from M2 macrophages could promote the invasion and migration of trophoblasts by activating the PI3K/AKT/Erk1/2 pathway (52). It can be inferred that PI3K/AKT is a pivotal pathway for intercellular communication. Furthermore, multiple hub-secreted protein-encoding genes, such as FGF9, THBS1, MET, etc. were all enriched in the PI3K/Akt signaling pathway. For instance, FGFs (fibroblast growth factors) are pleiotropic growth factors and is able to coordinate cell proliferation and differentiation, which has been proven to activate PI3K/AKT signaling pathways (53). Thrombospondin 1 encoded by THBS1 is an antiproliferative gene that can reduce the levels of PI3K and Akt (54, 55). Thus, we speculated that these secreted proteins communicated between cells at the maternal-fetal interface by activating multiple intracellular signaling cascades of recipient cells, which are the focus of future research.

In addition, Spearman correlation analysis was performed between the hub gene of recipient cells and hub-secreted protein-encoding genes from DSC, decidual macrophages, and the placenta. Results displayed that EFEMP1 and FGF9 derived from DSC were correlated with C3 and GBP1 in decidual macrophages FBLN5 derived from macrophage might be closely associated with TFPI2 in DSC. CD163 and SERPINA1 derived from placenta might be closely related to IFIT3 and OAS1 in decidual macrophages, respectively. Taking negatively correlated FGF9 and GBP1 as an example, FGF9 has been confirmed to promote M2 polarization in a variety of diseases (56, 57), At the same time, GBP1 has been verified to participate in M1 polarization in a variety of infectious diseases (56, 58), and is considered to be capable of regulating the polarity of M1/M2 macrophages in the tumor microenvironment (59). Based on existing studies, we speculated that the abnormal expression profilings of FGF9 secreted by DSCs and GBP1 in macrophages might lead to the imbalance of M1/M2 polarization at the maternal-fetal interface of URSA. However, how FGF9 affects GBP 1 and how they participate in the regulation of macrophage polarization is required to be further studied. In addition, other highly correlated molecules are also worthy of further investigation so as to elucidate the molecular mechanism of cell communication at the maternal-fetal interface of URSA.

As the traditional diagnosis of URSA relies on exclusion diagnosis after extensive screening, exploring new diagnostic strategies is imperative. Considering the potential of exocrine proteins as diagnostic markers, we explored novel diagnostic markers and diagnostic models for URSA by LASSO and SVM-RFE algorithms based on secreted protein-encoding genes in DEGs. Results revealed that 3 secreted protein-encoding genes (FGF9, IL1R2 and NID2) derived from DSC, 3 secreted protein-encoding genes (CFB, NID2 and CXCL11) derived from macrophages and 2 secreted protein-encoding genes (SFRP5 and SOSTDC1) derived from placenta exhibited remarkable diagnostic value which were confirmed by ROC. In addition, nomograms was constructed based on DSC and decidual macrophage-derived biomarkers, and the predictive performance and robustness of the model were also confirmed. Furthermore, we also performed validation in clinical samples by immunofluorescence co-localization analysis, which further confirmed FGF9, IL1R2, CXCL11, CFB and NID2 as biomarkers. Besides, consistent with our findings, CXCL11 and FGF9 have been verified to be valuable for the diagnosis of URSA (60, 61). As for IL1R2, CFB and NID2, studies have confirmed that IL1R2, encoding interleukin 1 receptor type 2 can bind to interleukin 1 and prevent its inflammatory actions, which then inhibit the signaling and influx of the inflammatory cascade (62). And a positive correlation between IL-lβ concentrations and successful pregnancy outcome in IVF have been reported (63). CFB, encoding complement factors B, can activate C3, thereby initiating the alternative pathway, leading to self-injury and is associated with the dysregulation of placental development (64). A study by Xue et al. confirmed that high serum CFB concentration during mid-pregnancy is a potential predictor of the risk of preeclampsia in patients with gestational diabetes mellitus (65). With respect to NID2, encoding idogen-2 may be a biomarker for decidualization initiation and development (66), while decidualization deficiency is an essential pathological feature of URSA. All the above studies have revealed the close relationship between the abnormal expression of these markers and pregnancy complications, however, there is a gap in the studies in URSA. Our study focuses for the first time on the diagnostic value of these molecules in URSA. More large-scale clinical studies are necessary to confirm their diagnostic performance. Taken together, this innovation undoubtedly provides new inspiration for the diagnosis and treatment of URSA.

## 5 Conclusion

**Figure 12:**
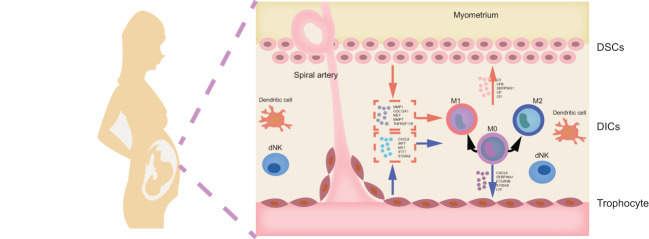
Mechanism hypothesis diagram. DICs, decidual immune cells; DSCs, decidua stromal cells.

In conclusion, our study is the first time to explore cellular communication between DSCs, macrophages, and placental trophoblasts at the maternal-fetal interface with an integrated research strategies based on bioinformatics analysis and experiments. This study signified that abnormalities in intercellular communication involving macrophages was a potential pathological mechanism of URSA, and the potential specific molecular mechanism is shown in **Figure 12**. In addition, this is the first time we utilize machine learning and experiment validation to screen URSA diagnostic markers. Taking FGF9, IL1R2, NID2, CFB and CXCL11 as examples, these molecules may be instrumental in the sensitive diagnosis of URSA. Taken together, all our findings confirm the involvement of cellular communication at the maternal-fetal interface in URSA and its close correlation with macrophages, which provide new insights into the pathogenesis, diagnosis and treatment of URSA.

## Data availability statement

The datasets presented in this study can be found in online repositories. The names of the repository/repositories and accession number(s) can be found in the article/**Supplementary Material**.

## Author contributions

XZ and HZ: conceptualization. XZ and YJ: data curation. YJ and HZ: data analysis. XZ, SL and YZ: writing-original draft. HZ: reviewed the manuscript. All authors read and approved the final article. All authors contributed to the article.

## Supplementary material

Supplementary Figure 1 Data preprocessing and normalization. The box plots of the GSE26787 and GSE165004 datasets before (A) and after (B) normalization. Three- dimensional PCA cluster plot of the GSE26787 and GSE165004 datasets before (C) and after (D) normalization.Red nodes represent GSE26787 and yellow nodes represent GSE165004.

Supplementary Figure 2 The receiver operating characteristic (ROC) curve of genes from the intersection of LASSO derived signature genes and SVM-RFE derived signature genes. **(A-C)**There signature genes in DSCs. **(D-F)** There signature genes in decidual Mφ. **(G-H)** Two signature genes in URSA placental.
