# Supplementary figures and images for: Intercellular communication involving macrophages at the maternal-fetal interface may be a pivotal mechanism of URSA: a novel discovery from transcriptomic data
Source: Front Endocrinol (Lausanne). 2023 May 17;14:973930. doi: 10.3389/fendo.2023.973930 (PMC10231036; doi:10.3389/fendo.2023.973930)

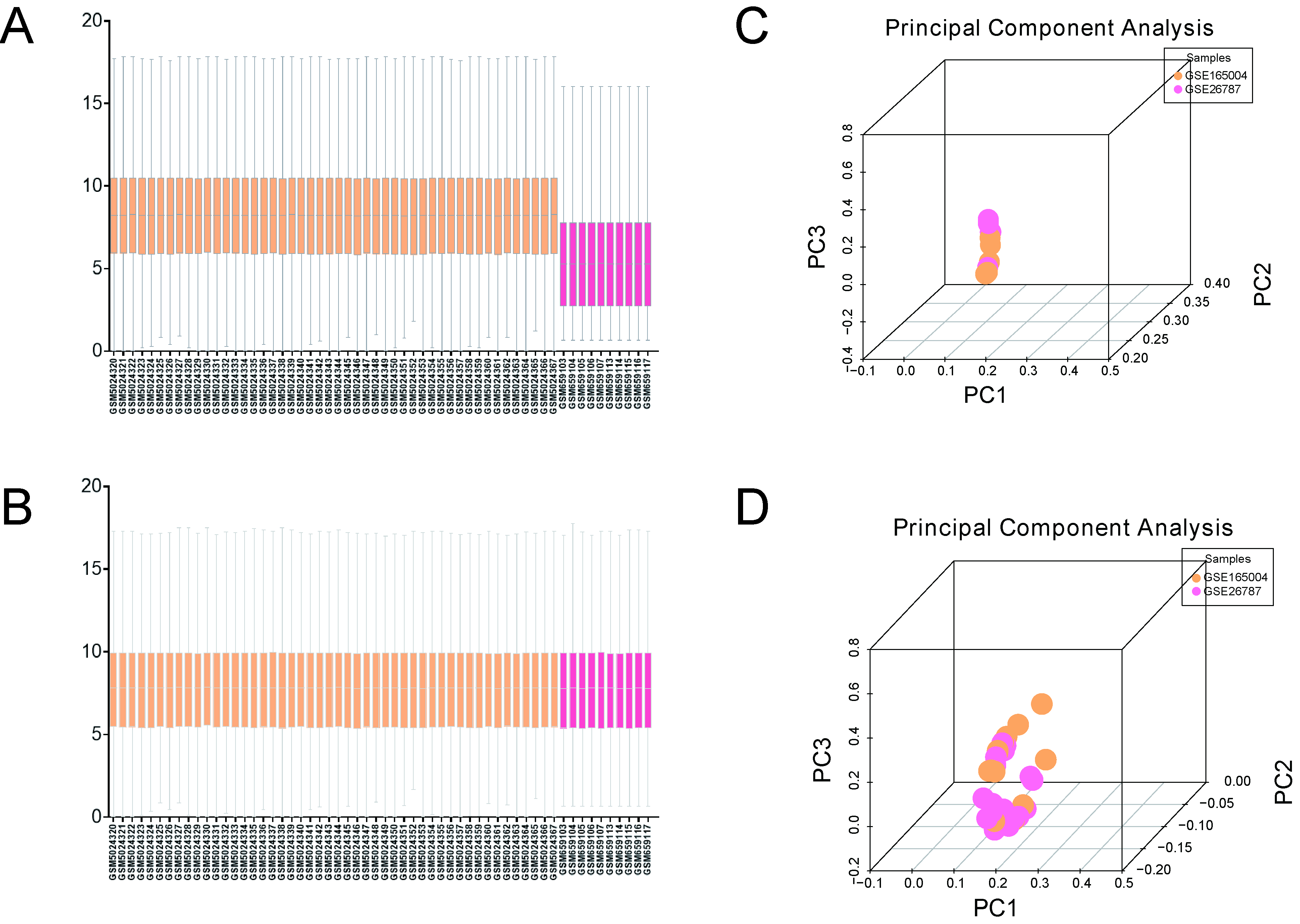

Supplement: Supplementary Figure 1 — Data preprocessing and normalization. The box plots of the GSE26787 and GSE165004 datasets before (A) and after (B) normalization. Three- dimensional PCA cluster plot of the GSE26787 and GSE165004 datasets before (C) and after (D) normalization.Red nodes represent GSE26787 and yellow nodes represent GSE165004. [file Image_1.tif]

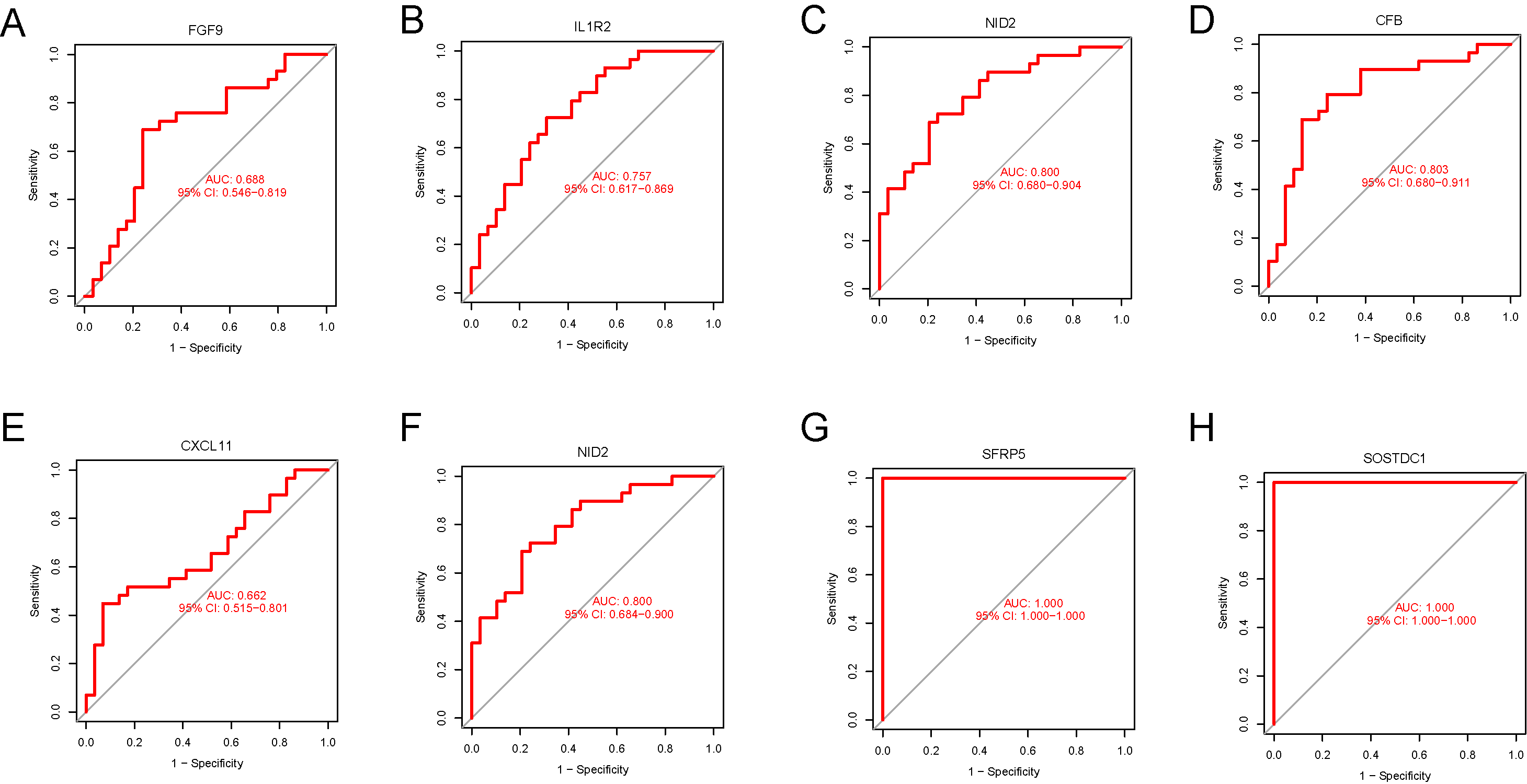

Supplement: Supplementary Figure 2 — The receiver operating characteristic (ROC) curve of genes from the intersection of LASSO derived signature genes and SVM-RFE derived signature genes. (A-C)There signature genes in DSCs. (D-F) There signature genes in decidual Mφ. (G-H) Two signature genes in URSA placental. [file Image_2.tif]
